# Assessment of occupational health and safety practices at government mortuaries in Gauteng Province: a cross-sectional study

**DOI:** 10.11604/pamj.2021.38.76.21699

**Published:** 2021-01-22

**Authors:** Mapula Luckyjane Molewa, Thokozani Patrick Mbonane, Joyce Shirinde, Daniel Masilu Masekameni

**Affiliations:** 1 Department of Environmental Health, Faculty of Health Sciences, University of Johannesburg, Johannesburg, South Africa,; 2 School of Health Systems and Public Health, Faculty of Health Science, University of Pretoria, Pretoria, South Africa,; 3 Division of Occupational Health, School of Public Health, University of the Witwatersrand, Johannesburg, South Africa

**Keywords:** Health, safety, hazards, risks, mortuary, practices

## Abstract

**Introduction:**

hospital mortuaries are responsible for the receipt and storage of deceased people. This exposes mortuary workers to a variety of health and safety hazards, which include physical, chemical, ergonomics, biological and psychosocial hazards/stressors. The aim of this study was to assess occupational health and safety practices (OHS) among government mortuary workers in Gauteng province.

**Methods:**

a cross-sectional descriptive study design was conducted between the year 2017 and 2018. A convenient sampling technique was used to sample 11 government hospitals in Gauteng Province. A total of 46 employees participated in the study. Data was collected using structured questionnaires and observational checklists. Ethical clearance and permission to conduct the study were obtained prior to the commencement of the study. Data was analyzed using Statistical Package for the Social Science (SPSS) version 25 software.

**Results:**

thirty-one (67%) of the respondents did not know the concept of hazard. Observations indicated that 5 out of 11 (45%) facilities were well maintained with only 2 (18%) of the facilities had the participants wearing the required PPE on duty. There was no association between working experience and having a knowledge of the existing hazards. However, there was a high correlation (P<0.05) between training and adherence to safe practices.

**Conclusion:**

the OHS practices were poor amongst operational employees. The study highlights the significance of developing and implementing Occupational Health and Safety programmes. We recommend that these programmes should focus on occupational health and safety education, training, supervision, medical surveillance and monitoring strategies must be developed and implemented.

## Introduction

Globally, more than 2.3 million premature deaths are recognized to be due to occupational environments; 2 million deaths result from occupational diseases, with the remaining being work-related occupational injuries [1]. Approximately 6400 workers are killed by occupational-related diseases, with 860 000 lives lost due to accidents at work daily [2]. Workers in the mortuary division of any hospital are involved with a number of activities, which includes the collection of corpses, transportation and temporary storage thereof. In some instances, post mortem and embalming activities are conducted at the corpse storage facility. In addition to operational sites, there are some sections such as waiting and viewing areas, which are accessible to the general hospital staff and public members. Shaha and colleagues identified six classes of occupational health and safety (OHS) hazards that potentially can have an impact in autopsy related practices [3]. These hazards include sharp objects, electrical, physical, biological, radiation and chemical hazards. Risk of exposure to mortuary related activities varied depending on exposure stressors. Poor OHS practices affect both the employer and workers in diverse ways [4]. The cost on employers varies, it may include medical expenses, vicarious liability as well as compensation. In addition to financial costs, it is an element of integrity damage and loss of production due to medical boarding of workers, or time spent away from the working environment attending to medical care [4]. On the side of the employee, the cost of occupational injury or disease could have a negative impact on production-related earnings and results in shorter working life span [5].

In South Africa, there are limited studies focusing on OHS practices among mortuary workers in a public health facility. Meel [6] conducted a risk assessment study in Umtata General Hospital´s mortuary, in Eastern Cape, in 1998. The aim of the study was to estimate the risks of exposure among mortuary workers, to determine the prevalence of major risk factors and to assess the effectiveness of the existing control procedure in place. The abovementioned study found that control measures implemented at the time were insufficient and unsatisfactory [6]. These findings suggest the need for additional studies in a related field in order to advance policy formulation and enforcement thereof. Hence, the aim of this study was to assess occupational health and safety practices amongst mortuary workers in Gauteng Province. While, the objectives to determine the level of knowledge, adherence to safety practices as well as identifying gaps which can influence health and safety practices amongst mortuary workers in Gauteng Province.

## Methods

**Study design:** a descriptive cross-sectional study was conducted between August 2017 and January 2018.

**Study population and sampling strategy:** a sampling frame in the study was mortuary facilities in the 33 government hospitals based in Gauteng Province, one of South Africa´s nine provinces. A convenience sampling was used to select 18 facilities. Two hospitals refused to participate and five did not respond; ultimately 11 hospitals participated in the study. The researcher further used purposive sampling to select the participants in the identified health facility (government hospital) of the study. Private undertakers were excluded from the study. A total of 46 questionnaires were completed.

**Data collection:** to achieve the aims of the study, a semi-structured questionnaire and an observation checklist were developed by the researcher with the assistance of the supervisors. The tools were formulated and base on the requirements of the Occupational Health and Safety Act (OHSA) 85 of 1993 and Environmental Health norms and standards [7, 8]. The questionnaire consisted of closed and open-ended questions which focused on determining the level of knowledge and adherence to health and safety practices. An observation checklist was completed for each facility and focused on identifying gaps which can contribute to unsafe practices in the mortuaries. Additional data was captured by the researcher who observed the participants while carrying out their duties at the study site.

**Data analysis:** data was captured onto a Microsoft Excel spreadsheet for coding and cleaning. Thereafter, it was transferred to the Statistical Package for Social Sciences (SPSS), version 25 for analysis. Data analysis was conducted with the assistance of a biostatistician. Tables were used to present basic descriptive analysis such as means, percentages and frequencies for the categorical variables. Inferential analyses was kept minimal, only cross tabulation was used for associations and the Fisher´s exact test was used to analyse the contingency tables.

**Ethical considerations:** the study was granted ethical approval by the University of Johannesburg: Faculty of Health Sciences Research Ethics Committee (REC-01-91-2017). We received permission to conduct the study and approach government hospitals from the Gauteng Department of Health. The management of each hospital gave us permission to access and recruit the participants. Participant gave written consent before participating in the study.

## Results

### Questionnaire

**Demographic information:** forty-six respondents participated in the study, 18 females (39%) and 28 male respondents (61%). Most participants (43%) were in the age group of 45 to 65 years, and there were 13 (28%) in each of the 18 to 35 years and 36 to 44-year age groups. Half of the respondents had Matric (50%), 15 (33%) had grade 11 and 6 (13%) had a certificate; a small number of participants 2 (4%) had a Baccalaureate degree. Among the participants, 15 (33%) were auxiliary workers, 13 (28%) were admin clerks, 12 (26%) were porters and 6 (13%) were cleaners.

**Knowledge of workplace health and safety hazards:** thirty-one (67%) of the respondents did not know the concept of a hazard. Thirty-three (33%) respondents had different opinions and perceived it as something that can cause injuries, danger and or cause diseases as indicated in Table 1. There was no association between working experience and having knowledge of the existing hazards. Three (8%) of the 36 participants identified some of the hazards as odour, 1 (3%) Healthcare Risk Waste HCRW, 3 (8%) violent clients or bereaved families, 6 (17%) sharps, 2 (6%) body fluids and 21 (58%) infectious agents.

**Table 1:** definition of hazard; knowledge of existing hazards vs health outcomes

Variable	Frequency	Percentage
**Definition of hazards**		
Causes injuries/danger	4	9%
Causes diseases	11	24%
Did not know	31	67%
**Knowledge on existing hazards**		
Know about hazard	36	78%
Do not know	10	22%
**Contribution of working environment towards personal health problems**		
Yes	29	63%
No	17	37%
**Participants diagnosed with occupation medical condition**		
Yes	25	54%
No	21	46%

Thirty-six participants (78%) perceived infectious diseases as a high-risk exposure, four participants (9%) found it to be low risk and six (13%) as no risk. Working conditions were rated as the second-highest risk exposure by 25 participants (54%), 18 (39%) rated manual handling, trips, slips or falls was counted as high-risk exposure by 13 (28%), and awkward posture and chemical exposure were rated high risk by 10 (22%) participants. Most participants (76%) perceived working hours (long shifts) as no risk to their health and safety, 21 (46%) chemical exposure, 18 (39%) awkward posture, 11 (24%) blood exposure, 11 (24%) for manual handling and infectious diseases, whilst 25 (54%) perceived slips, trips or falls as having no risk towards their well-being at the workplace. Twenty-nine participants (63%) reported working conditions did contribute to their personal problems, whereas 17 (37%) said the work conditions could not affect them. Working conditions included facilities, equipment and materials used at work.

In order to cope with stressful working conditions, 16 (32%) participants from the study population consulted with the Employee Health and Wellness (EHW) programme, 5 (11%) with supervisors, 4 (9%) absented from work and 4 (9%) smoked. Few participants 2 (4%) drank alcohol and only 3 (7%) would become aggressive at work; 15 (33%) confirmed to do nothing. Those who consulted with EHW listed other measures, such as visiting private doctors, human resources, talking with colleagues as well as consulting with their religious groups.

On the high-risk rating scale of contracting occupational diseases, 34 (74%) participants were concerned about being at high risk of contracting occupational Tuberculosis (TB), 22 (48%) Hepatitis B or C, 21 (46%) Meningococcal Meningitis, 19 (41%) Septicaemia and 17 (37%) Human immunodeficiency virus (HIV). Twenty participants (43%) were not at all concerned about contracting HIV at the workplace, 18 (39%) Meningococcal Meningitis, 19 (41%) Septicaemia, 11 (24%) Hepatitis B or C and five (11%) TB. Twenty-five participants (54%) disclosed they were once diagnosed with a medical condition related to work and 21 (46%) said they were never diagnosed. About 25 (54%) of the study population sustained an occupational medical condition. Amongst the 25 participants, 16 (64%) sustained occupational injuries and the remaining occupational diseases.

**Figure 1:**
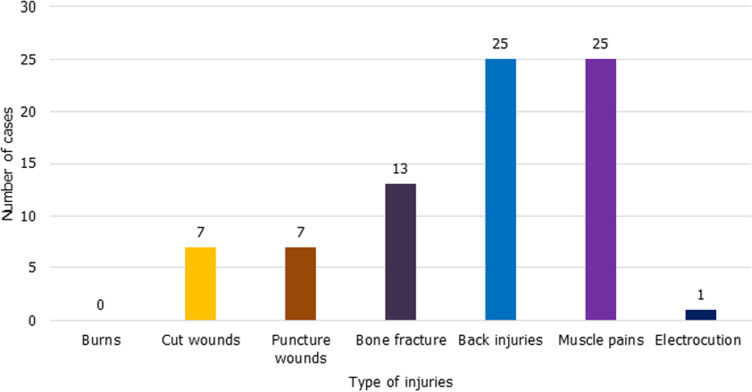
sustained occupational injuries in the mortuary

According to Fisher´s Exact Test, there is no significance between gender and the likelihood of being diagnosed with an occupational medical condition associated with working in the mortuary (p= 0,432). However, a little difference was illustrated as 20 (71%) males were diagnosed with an occupational medical condition compared to the 10 (56%) females. When classifying the type of medical conditions by gender, 12 out of 20 (75%) males sustained injuries compared to 4 out of 10 (40%) females. Eight out of 20 (40%) males and 7 out of 10 (70%) females reported for occupational disease. Figure 1 indicates the types of injuries sustained by the participants in the mortuaries while performing their functions. From the data, the muscle pains and back injuries were found to be the most common incidents as compared to other safety-related injuries. Furthermore, about 57% of the study population alluded that unsafe environmental conditions are the leading risk factors in causing physical bodily harm.

**Knowledge of transmission routes of infections:** thirty-nine participants claimed to know the routes of exposure. One (2%) identified inhalation, 1 (2%) ingestion, 6 (13%) cuts or abrasion and 16 (35%) identified skin contact as route of exposure. The seven participants who did not know the routes of exposure, three (43%) were diagnosed with a medical condition.

**Vaccination:** all the respondents thought it was important to be vaccinated. About 30 (65%) participants have been vaccinated during their course of employment, whereas 16 (35%) have never been vaccinated in their current employment. The Fisher´s Exact Test had a p-value of 0.063, which is larger than 0.05, thus there is no correlation between the years of working experience and the likelihood of being vaccinated. The type of vaccination varies depending on the type of microbiological agents and legislative requirements associated with a specific occupational activity. Most of the participants 17 (57%) had been vaccinated against Hepatitis C, 5 (17%) Hepatitis B, 1 (3%) Meningococcal Meningitis, 1 (3%) Tetanus, 1 (3%) all the mentioned vaccines, 4 (13%) were unsure of the type of vaccine received. Twenty-one participants had gone for pre-medical surveillance before employment at their current workplace and 25 (54%) never went. Half of the participants 27 (59%) never went for continuous medical surveillance, 14 (30%) attended yearly and 5 (11%) underwent after more than 5 years.

**Adherence to health and safety:** forty participants (87%) alleged to be knowing the systems of reporting occupational incidences or accidents, 2 (4%) unsure and 4 (7%) did not know them. Eighteen participants (39%) claimed to have access to the summary of OHSA 85 of 1993, 26 (57%) did not have any access, and 2 (4%) unsure. Approximately 36 (78%) participants claimed to have standard operating procedures (SOPs) available at their workplace, 7 (15%) did not have and 3 (6%) were unsure. Twenty-nine participants (63%) had received training on SOPs, whilst 17 (37%) had never been trained. Thirty-four (74%) participants claimed to follow the SOPs, 2 (4%) were not sure and 10 (22%) confirmed to not follow them. All the participants who received training on SOPs followed them, whereas 10 (67%) who did not receive training did not follow the SOP. According to Fisher´s Exact Test, p = 0.00, which is less than 0.05, Phi=0.754 is greater than 0.5 large effect, thus there is a high correlation between training and adherence.

Twenty-six participants (57%) did not receive Personal Protective Equipment (PPE) from the employer and used their own personal clothes; about 20 (43%) were provided with PPE by the employer. It was observed that the participants (100%) had facemasks readily available for use, 45 (98%) had surgical gloves, 27 (57%) cleaning plastic aprons and 24 (52%) waterproof footwear. Few participants (39%) had safety glasses, 16 (34%) cut resistant gloves, 14 (30%) surgical shirts and trousers, only 10 (22%) were provided with long-sleeved gowns. Fit test on PPE was done for the 11 (24%) participants. Less than half the participants (48%) used PPE every time, 13 (28%) sometimes and 12 (26%) never use it. Majority of participants 27 (59%) never took PPE home, 16 (35%) took it every time and three (7%) took it sometimes. There were few challenges associated with the use of PPE. Nine participants (20%) had allergies towards latex, 4 (7%) complained about the sizes and 16 (35%) lacked storage for the PPE. Eleven participants identified other challenges such as staff sharing PPE (2%), 1 (2%) PPE being of poor quality and 9 (20%) confirmed delay in procurement. However, 6 (13%) did not mention any challenges with regards to the use and provision of PPE. Many participants (80%) claimed to their SHE Representatives and 9 (20%) did not. Half of the participants (50%) did not have meetings, five (11%) attended weekly, three (7%) monthly, eight (17%) quarterly and seven (15%) met yearly.

**Sources of information:** few participants (33%) conducted safety talks to visitors or clients, including any staff member that visited their mortuary facilities, whereas 31 (67%) participants do induct the visitors. Twenty-three participants (50%) received information from OHS or EHW staff nurses, 20 (44%) from Environmental Health Practitioners (EHPs), 3 (6%) from other sources such as Safety, Health, Environmental (SHE) Representatives, Human Resource Management (HRM) or supervisors.

**Summary of observational checklist:** the summary of the observations indicates that 5 (45%) facilities have their participants` PPE well-maintained and 2 (18%) have their participants wearing the required PPE on duty. The frequently used PPE set was observed to be facemasks and surgical gloves. Storage places for PPE were provided to 8 (73%) of the facilities. Five facilities (45%) were well maintained structurally, with well-functioning ablution facilities as well as proper safety signages. Three facilities (27%) had a foul smell. Trolleys for transporting corpses were adjustable, clean and in good working conditions in 7 (64%) facilities. Only 2 (18%) of the facilities segregated the infectious from non-infections corpses in storage as well as storing chemicals safely. Temporary storage for waste was kept in good order in 7 (64%) of the facilities. None of the facilities (100%) had evacuation plans available. A summary of the OSHA 85 of 1993 was seen at 1 (2%) facility.

## Discussion

Although the dead are not regarded as being a health hazard, mortuary workers are at increased risk of occupational hazards when handling the bodies [9]. The level of knowledge on health and safety was not satisfactory as the participants were unable to conceptualize the concept of a hazard. It was proven throughout the study that the term was related to something that caused disease rather than an injury. Bakhshi [10] suggests a safe working environment is the greatest safeguard against infections. The fact that half of the participants perceived working conditions, environmental factors and human factors do not contribute to workplace accidents, an assumption can be drawn that nearly a half of participants remain at risk of being exposed to hazards. Participants could at least list six workplace hazards existing within their working environment. Even when the participants were provided with the list of different exposures and asked to rate the risk of exposure, there was a lack of awareness on ergonomic and chemical hazards in comparison with biological hazards. This could be because the participants were fully aware that most corpses carry different pathogens. This was the first study to identify electrocution as one of the sustained injuries. The results however, did show similarity with the study in Irish about burn injuries as there were no reported cases [11, 12]. TB was found to be the most rated disease of concern. This was because the most known common route of exposure for TB was found to be through airborne (inhalation) and because the disease has been a well-known burden in the healthcare industry and to the public in general.

According to reports, low literacy levels have been associated with a high incidence rate [13]; the statement may be true as in this study the literacy level remained low and the reported cases remained high. Institutions need to develop a clear training curricular for mortuary workers, which will also include OHS content. Although the participants used EHW services and consulted at the staff clinic, other participants preferred private services. It was that: *"the internal healthcare practitioners do not regard their illness as work-related and perceive the participants seeks a token of absenteeism, hence the use of private consultation"*. This statement indicates there are poor advocacy and awareness towards the mortuary workers regarding EHW services and that there is a need to for EHW practitioners to reach out to the employees in the healthcare sector. An emerging issue shows that OHS challenges do not only contribute to contracting occupational diseases or sustaining injuries but also can have an impact on the morale, health and productivity of employees. This study revealed that they already account for 9% of absenteeism in the study population. They also have an influence on behavioural change, which shifts from healthy behaviour to unhealthy, such as becoming aggressive and addictiveness to smoking. The results revealed that the most likely workers to be vaccinated during their employment are those with less than 5 years´ experience as compared to the ones with above 5 years. This can be due to some hospitals having just introduced the vaccination programme and other managers mentioned they are yet to develop a programme for vaccination of high-risk employees. Most participants were vaccinated against Hepatitis and few were not sure of the type of vaccines they had received. The OHSA 85 of 1993 requires that workers be taken for medical surveillance before or within 14 days after commencement of employment where any exposure exists, however poor conformance was noted as well as on the frequency of undergoing for periodic assessment [7].

The results of this study suggests that mortuary attendants are without formal education, which might be a crucial risk factor to have basic knowledge of safe practices and behaviour [14]. Similarly, a study in Bangladesh found that none of the workers had received training in any practice of mortuary care [12]. Lack of training on SOPs in this study resulted in participants being not sure if they are following the correct procedures and the others declaring not to be following the SOPs. This could be because they do not understand how to interpret the content of the SOPs and also do not have a basic background on safe practices in the mortuary environment. Mittal [15] suggests that people who are literate can successfully understand SOPs as well as adhering to health and safety practices; this was true because most who were trained have followed the procedures. Hence, the study also found a strong association between training and adherence to safe work practices. Safety induction such as the proper PPE to be worn and rules when entering the storage area in case of private undertakers or family members who want to collect the bodies were less likely communicated. OHS practitioners were the main communicators of OHS issues in most facilities. This was because most workers in the staff clinic are OHS nurses and are able to interact and advise workers during sites visit or consultation. The study was able to identify that most facilities do not provide participants with essential PPE. The participants who were not provided with PPE resorted to using their own personal clothing and took it home with. It was observed that the lack of storage for PPE also influenced participants to take their PPE home with. Gaps influencing the proper usage of PPE included allergic reactions towards latex, sharing of PPE, delayed procurement, and poor fitting sizes.

The findings in this study were consistent with a study in Nigeria, which described mortuaries as abandoned and not considered as a priority in resource allocation [16, 17]. The design and the environmental factors such as the availability of adequate ventilation, flooring, furniture and sewage disposal as well as odour control are very important to ensure safety and adherence to the SOPs [3]. In this study, more than half of the facilities had debilitated structures, stained floors, peeling walls and ceilings, some of the toilets were not functioning. A foul smell was observed which was due to malfunctioning freezers for storage as well as an inadequate ventilation system. The observed malfunctioning trolleys can be a source of exposure to back injuries and back pains as they required more effort to pull and operate. Some of the trolleys had exposed sharp edges that could results in punctures and cuts if not properly handled. Other facilities had designated corpse storage areas and labelled them for infectious and non-infectious diseases which are a practice to minimize an incident of accidental exposure to communicable diseases. Likewise, the practice of segregation assists workers in taking the required precautions depending on the type of the disease. Due to insufficient designated storage areas, personal belongings of the workers and some chemicals were stored together.

This study identified gaps in the OHS knowledge, practice and implementation of OHS within government mortuaries. Hence, we recommend future research/studies on the following areas: investigating the prevalence and incident of occupational diseases and injuries; compliance of mortuary services to environmental health norms and standards; the effectiveness of OHS policies or control measures in the mortuary workplace; the impact of capacity on the health and safety practices, the impact of temperature on the health of employees, surface contamination in the mortuary; quality of air or efficiency of the ventilation system in the mortuaries, as well as the efficiency of the PPE used by the mortuary workers.

## Conclusion

Occupational health and safety objectives are to ensure the protection of employees, the public and the environment from the detrimental effects that can arise from working activities. Government mortuaries, as temporary storage for the deceased, harbor all kinds of hazards, which through literature are proven to possess properties that may cause allergy, toxicity or harm to health and safety. Several literatures as referenced in the main body of the manuscript have shown that there is paucity of information on occupational exposure in mortuary settings, especially in developing countries. The summary of the study indicated that harm could occur to both the workers and employers through the loss of time and production due to uncontrolled occupational conditions. As such, mortuary workers in Gauteng Province government mortuaries are not exempt from complying with OHS practice. This study found that workers do have a fair knowledge of OHS but do not adhere to safe work practices. There are gaps identified in the study which may lead to poor practices, and the main gaps include lack of training on workplace safety procedure, provision and maintenance of PPE, as well as ensuring continuous medical surveillance in the workplace. Just as in any profession, some of the risks can be prevented, eliminated, minimized and/or controlled.

### What is known about this topic

- Current health and safety mitigatory measures in mortuaries are insufficient;
- There is no formal education relating to safe mortuary practices and behaviour;
- An unsafe work environment contributes to the incident of acquiring occupational diseases and sustaining injuries on duty.

### What this study adds

- Despite the lack of formal training, a number of gaps are identified which contributes to poor health and safety practices;
- OHS challenges also have an impact on the morale, health and productivity of employees;
- Advocacy and awareness on utilization of Employee Health and Wellness Services rendered by employers must be strengthened among mortuary workers and other healthcare practitioners.
